# Simultaneously Improved Thermal and Dielectric Performance of Epoxy Composites Containing Ti_3_C_2_T_x_ Platelet Fillers

**DOI:** 10.3390/polym12071608

**Published:** 2020-07-19

**Authors:** Lin Chen, Yu Cao, Xuebo Guo, Ping Song, Kai Chen, Diansen Li, Jun Lin

**Affiliations:** 1 MOE Key Laboratory of Power Station Energy Transfer Conversion and System, School of Energy Power and Mechanical Engineering, North China Electric Power University, Beijing 102206, China; 13264243432@163.com (Y.C.); 120192202712@ncepu.edu.cn (X.G.); 1182202225@ncepu.edu.cn (P.S.); 2 MOE Key Laboratory of Enhanced Heat Transfer and Energy Conservation, School of Chemistry and Chemical Engineering, South China University of Technology, Guangzhou 510640, China; chenkaihb09@126.com; 3 MOE Key Laboratory of Bio-Inspired Smart Interfacial Science and Technology, School of Chemistry, Beijing University of Aeronautics and Astronautics, Beijing 100191, China; lidiansen@buaa.edu.cn; 4 School of Renewable Energy, North China Electric Power University, Beijing 102206, China

**Keywords:** MXene, Ti_3_C_2_T_x_, polymer composites, thermal conductivity, dielectric properties

## Abstract

Polymer composites with enhanced thermal and dielectric properties can be widely used in electric and energy related applications. In this work, epoxy composites have been prepared with Ti_3_C_2_T_x_, one of the most studied MXene materials that can be massively produced by direct etching using hydrofluoric acid. The addition of conductive two dimensional Ti_3_C_2_T_x_ platelet fillers leads to improved but anisotropic thermal conductivity of the composites. The through-plane thermal conductivity reaches 0.583 Wm^−1^K^−1^ and the in-plane thermal conductivity reaches 1.29 Wm^−1^K^−1^ when filler content is 40 wt% (21.3 vol%), achieving enhancements of 2.92 times and 10.65 times respectively, as compared with epoxy matrix. The dielectric permittivity of epoxy composite is enhanced by a factor of ~2.25 with 40 wt% fillers, and the dielectric losses are within a small value of 0.02. The results prove the effectiveness of Ti_3_C_2_T_x_ in simultaneously improving thermal and dielectric performance of epoxy composites, and it is deduced that further improvements may be obtained by using Ti_3_C_2_T_x_ nanoflake fillers.

## 1. Introduction

Polymer composites with substantially improved thermal conductivity (*k*) [1,2,3] and dielectric permittivity (*ε*) [4] are in great need for many applications, such as thermal interface materials [5,6], heat dissipation and thermal management [7,8,9], microelectronics, and energy storage related applications [4,10,11,12]. Appropriate fillers with inherent high performance are essential for preparing these desired functional polymer composites. These days, a new class of two dimensional (2D) materials named MXene, which is often fabricated by selectively etching element A from “M_n+1_AX_n_” (M is an early transition metal, A is an A-group (mostly groups 13 and 14) element, X is carbon and/or nitrogen, and n = 1, 2, or 3) [13], receives a considerable amount of research attention [14]. To date, more than 20 MXenes have been synthesized [15] and the most studied one is Ti_3_C_2_T_x_ (T_x_ stands for the surface terminations of functional groups) [16], which has prospective applications in capacitors [17,18], electromagnetic shielding and absorption [19,20], water purification [21], and antibacterial activity [22].

In fact, Ti_3_C_2_T_x_ is also expected to be an ideal filler for preparing polymer composites with enhanced thermal and dielectric properties. Firstly, Ti_3_C_2_T_x_ is a good electrical conductor [13,14,23], which helps to improve composite thermal conductivity and dielectric permittivity. Secondly, the laminated structure of Ti_3_C_2_T_x_ means large aspect ratio, which may result in high thermal conductivity [24] and good dielectric permittivity [4] by percolation. Thirdly, the abundant functional groups on Ti_3_C_2_T_x_ surface indicate possible good compatibility with polar polymers when used as fillers, which would lead to enhanced composite properties. When thermal conductivity is improved simultaneously with dielectric permittivity, the better heat dissipation associated with higher thermal conductivity can result in a more stable dielectric performance. Thus, using Ti_3_C_2_T_x_ as fillers may achieve a synergistic effect between the improved thermal and dielectric performance of polymer composites, potentially highly beneficial to the dielectric materials, because they always face the problem of the temperature rise caused by dielectric loss, which may eventually lead to material breakdown if the heat is not effectively dissipated.

In order to verify the above expectations and evaluate the effect of Ti_3_C_2_T_x_ on thermal and dielectric performance, the widely used epoxy resin [25,26,27] was chosen as the matrix and Ti_3_C_2_T_x_/epoxy composites were prepared. According to our previous study on Ti_3_C_2_T_x_ MXene films [28], there are roughly three forms of Ti_3_C_2_T_x_, including Ti_3_C_2_T_x_ micro-platelets/particles resulted from hydrofluoric acid (HF) etching of Ti_3_AlC_2_ (herein, termed as HF-Ti_3_C_2_T_x_) and its derivants of un-delaminated multi-layer Ti_3_C_2_T_x_ nanoflakes (ML-Ti_3_C_2_T_x_) and delaminated few-layer Ti_3_C_2_T_x_ nanosheets (FL-Ti_3_C_2_T_x_), as shown in Supplementary Figure S1. In this work, the HF-Ti_3_C_2_T_x_ filler was used due to its high yield during the preparation process of Ti_3_C_2_T_x_. Moreover, the results of HF-Ti_3_C_2_T_x_/epoxy composites could set a benchmark for further work on composites prepared with the other two nanosized Ti_3_C_2_T_x_ fillers. The thermal and dielectric performance of the HF-Ti_3_C_2_T_x_/epoxy composites were analyzed and correlated with their structure and morphologies. 

## 2. Materials and Methods 

### 2.1. Materials

Ti_3_AlC_2_ powder (98% purity) was supplied by 11 Technology Co., Ltd., China. Hydrofluoric acid (HF, 40 wt%) and ethanol were produced by Beihua Fine Chemicals, China. Epoxy resin (E-51) was produced by Sanmu Chemical Co., Ltd., China. Curing agent (3-methyl-tetrahydrophthalic anhydride, MeTHPA) and cure accelerator (2,4,6-Tris(dimethylaminomethyl)phenol, DMP-30) were purchased from TCI (Shanghai) Development Co., Ltd., China. All materials were used as received. 

### 2.2. Preparation of HF-Ti_3_C_2_T_x_ and Epoxy Composites 

HF was used to selectively etch the Al in Ti_3_AlC_2_ to obtain Ti_3_C_2_T_x_ via the procedures described in literature [15,28,29]. Briefly, 2.5 g Ti_3_AlC_2_ powders were slowly added to 60 mL HF which was contained in a plastic beaker. The mixture was magnetically stirred at 480 rpm and 40 °C for 24 h. In this way, the Al was selectively etched, turning the Ti_3_AlC_2_ into HF-Ti_3_C_2_T_x_. The mixture was diluted by deionized (DI) water and centrifuged at 3500 rpm for 5 min to obtain the precipitate. Then, the procedures of washing precipitate and centrifugation to separate the precipitate were repeated until the pH value of supernatant was 7. The aqueous dispersion of HF-Ti_3_C_2_T_x_ was vacuum filtered to obtain the filter cake, which was washed by ethanol and then dried at 60 °C for 24 h, and the yield is ~70 wt%. The dried filter cake was later milled and used as fillers in epoxy composites. 

Epoxy resin and curing agent (MeTHPA) with mass ratio of 100:85 were mixed with certain amount of HF-Ti_3_C_2_T_x_ fillers (5~40 wt%). The mixture was magnetically stirred for 2 h, followed by ultrasonication for 10 min and degassing for 10 min. Afterwards, a cure accelerator (DMP-30, mass ratio of epoxy and accelerator is 100:1) was added to above mixture, which was successively stirred, ultrasonicated and degassed for a total 30 min (10 min for each process). Then, the composite fluid was slowly poured into pre-heated molds, which were manually inclined to enhance the composite fluid to flow around to fill the mold. After that, the composite fluid was cured at 100 °C for 4 h and 150 °C for 10 h. The resulting epoxy composites were circle plates (diameter of 25.4 mm) and square plates (40 × 40mm) with thickness of ~0.5mm. 

### 2.3. Characterization and Measurements

Scanning electron microscope (SEM) images of fillers and composites were taken by Quattro-S (Thermo Fisher Scientific, USA). Small fragments were mechanically fractured from the as-prepared epoxy composite plates by using tweezers, and the resulting cross sections were used for SEM observation. X-ray diffractometer (XRD) analysis was performed on SmartLab SE (Rigaku, Japan) under 40 kV and 30 mA with a scanning speed of 2°/min from 3° to 70°. Static water contact angles of the composites were measured by a telescopic goniometer (OCA15 EC, Dataphysics, Germany) by the sessile drop method. Thermal gravimetric analysis (TGA) was carried out on a thermal analyzer STA 449F5 (NETZSCH, German) under argon (Ar) atmosphere at a heating rate of 10 °C/min from 40 to 800 °C. Thermal diffusivity (*α*) was measured by a laser flash method using an LFA 467 (NETZSCH, German), which can measure both through-plane and in-plane thermal diffusivities via applying corresponding specified sample holders. Specific heat capacity (*c_P_*) was measured by differential scanning calorimetry method using STA 449F3 (NETZSCH, German) with a heating rate of 2 °C/min. Density (*ρ*) was measured by the buoyancy method using MH-300A (Qunlong, China). Then the thermal conductivity (*k*), was obtained by *k*=*α∙ρ∙c_p_*. Dielectric properties were measured by a precision impedance analyzer WK-6510B (Wayne Kerr, UK) within the frequency range of 10^2^–10^6^ Hz at room temperature. 

## 3. Results

**Figure 1:**
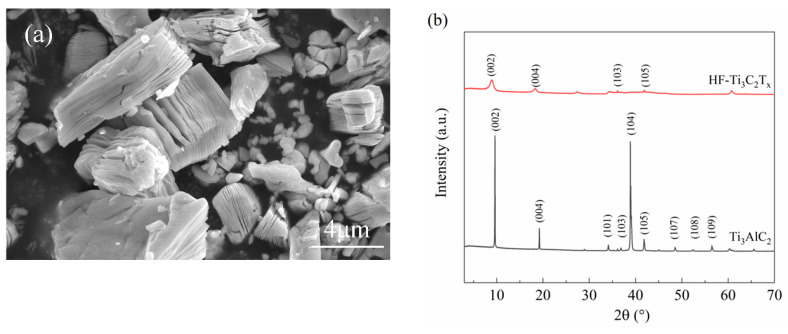
Characterization of HF-Ti_3_C_2_T_x_ fillers, (**a**) SEM image, (**b**) XRD patterns.

Figure 1a shows the accordion-like HF-Ti_3_C_2_T_x_ resulted from HF etching of Ti_3_AlC_2_ and the lateral dimension of HF-Ti_3_C_2_T_x_ is around 5~10 μm. Similar morphologies were also observed in literature [15,28,29], which is regarded as an indication of successful removal of Al layers in Ti_3_AlC_2_. The results of XRD tests on Ti_3_AlC_2_ and HF-Ti_3_C_2_T_x_ powders are shown in Figure 1b. For HF-Ti_3_C_2_T_x_ powders, the characteristic (104) peak at ~39° disappeared and the (002) peak at ~9.8° shifted to the lower angle, as compared with Ti_3_AlC_2_ powders. These results agree well with those reported in references [15,28,29], which further confirms the effective etching of Al. 

**Figure 2:**
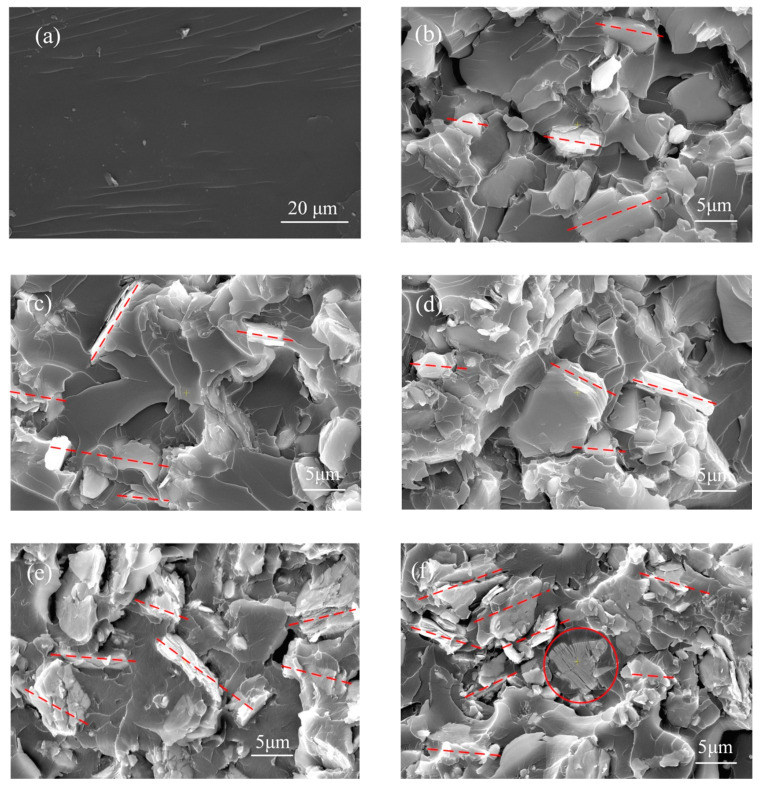
SEM images of cross sections of (**a**) Pure epoxy, (**b**–**f**) HF-Ti_3_C_2_T_x_/epoxy composites with filler content of 5 wt%, 10 wt%, 20 wt%, 30 wt% and 40 wt %, respectively.

Figure 2a is an SEM image showing a smooth cross section of pure epoxy, in which the wavy patterns are resulted from mechanical fracture that is used to obtain the cross section of samples. Figure 2b–f are SEM images of cross sections of HF-Ti_3_C_2_T_x_/epoxy composites with different filler contents. In general, the HF-Ti_3_C_2_T_x_ fillers, which are stack shaped platelets with relatively transparent and light color in the SEM images, are dispersed quite well in epoxy. The interface of the filler and the matrix is compact, as indicated by the circle (Figure 2f) showing fillers fully embedded in the epoxy matrix. This good interfacial compatibility may reasonably be attributed to the abundant functional groups (OH, F, O, H, etc.) on the HF-Ti_3_C_2_T_x_ surface [14], which improve the interfacial adhesion through the possible formation of chemical bonding between filler and epoxy matrix. For the low filler content of 5 wt% or 10 wt%, the platelet HF-Ti_3_C_2_T_x_ fillers are mainly surrounded by epoxy matrix and isolated from each other, as shown in Figure 2b,c. When the filler content increases from 20 wt% to 40 wt%, although the fillers still disperse homogenously in the matrix, the distance between fillers decreases and interconnections among fillers gradually appear, as shown in Figure 2d–f. From Figure 2b–f, it is also found that the platelet fillers tend to distribute along the in-plane direction, as indicated by the dashed lines for filler and filler chains.

**Figure 3:**
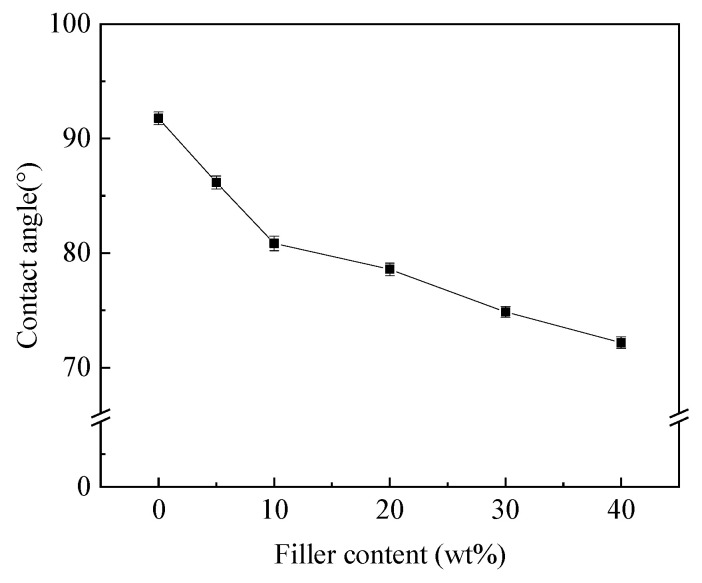
Static water contact angles of the HF-Ti_3_C_2_T_x_/epoxy composites.

Figure 3 shows the measured static water contact angle for above epoxy and epoxy composites, which decreases from ~92° of pure epoxy to ~72° of composite with 40 wt% filler, indicating increased hydrophilicity with higher filler content. This phenomenon can be correlated with the various hydrophilic terminals on the HF-Ti_3_C_2_T_x_ surface, which are also helpful for good compatibility between filler and epoxy matrix, as revealed by the SEM images in Figure 2. 

**Figure 4:**
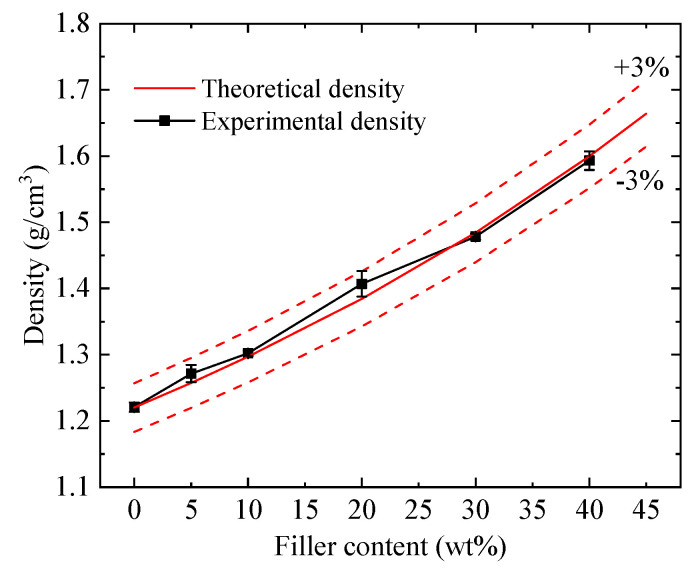
Density of HF-Ti_3_C_2_T_x_/epoxy composites.

Figure 4 shows the density of HF-Ti_3_C_2_T_x_/epoxy composites as a function of filler content, along with the theoretical density calculated by Equation S1 with epoxy density of 1.22 g/cm^3^ and HF-Ti_3_C_2_T_x_ density of 3.00 g/cm^3^ (data from 11 Technology). Generally, the experimental results agree well with the theoretical one, i.e., the deviations are within ±3%. Such results suggest that the interfacial compatibility between the filler and the polymer matrix is relatively good and free of voids, which is also confirmed by the SEM results discussed earlier. For the benefit of the broader scientific community, the weight percentage of HF-Ti_3_C_2_T_x_ filler in the epoxy composites can be converted into volume percentage by using Equation (1) [30],

(1) $$V_{f}=\frac{W_{f}/\rho_{f}}{W_{f}/\rho_{f}+\left(1-W_{f}\right)/\rho_{m}}=\frac{\rho_{m}W_{f}}{\rho_{m}W_{f}+\rho_{f}(1-W_{f})}$$

where $V_{f}$ and $W_{f}$ are the volume percentage and weight percentage of HF-Ti_3_C_2_T_x_ filler, respectively. $\rho_{m}$ and $\rho_{f}$ are the densities of epoxy matrix and HF-Ti_3_C_2_T_x_ filler, respectively. 

**Figure 5:**
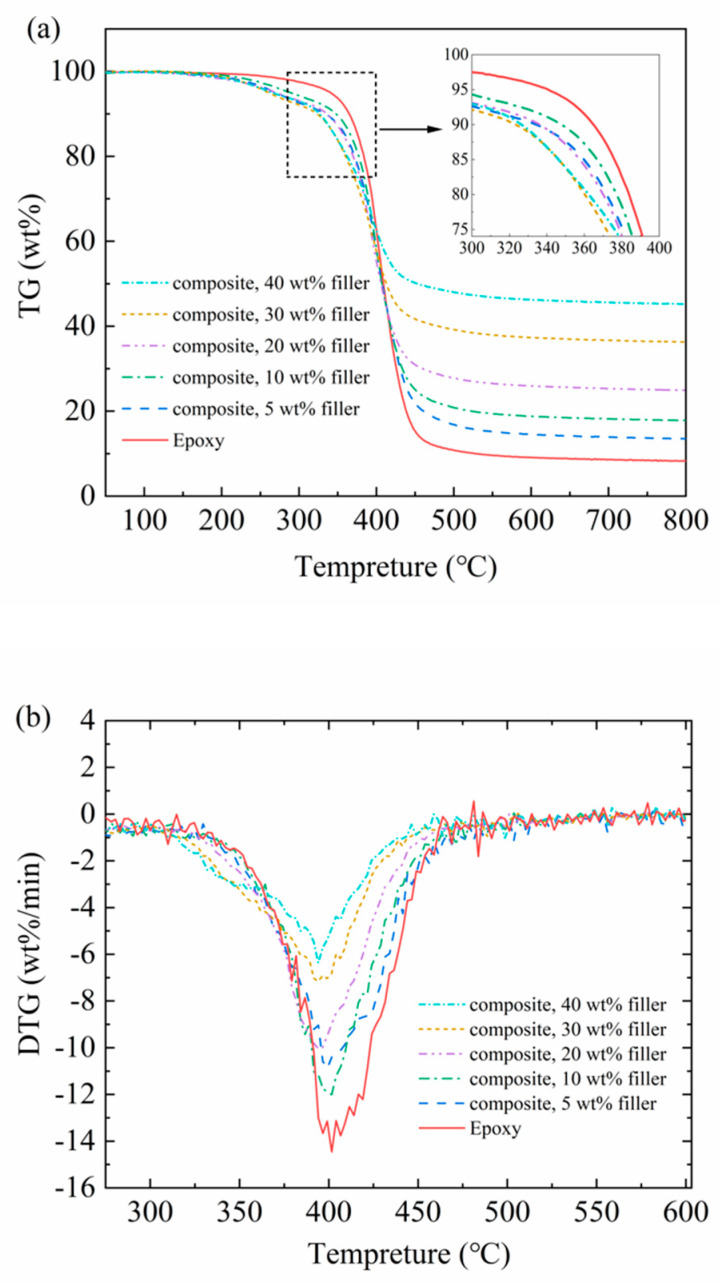
Weight loss curves of epoxy and HF-Ti_3_C_2_T_x_/epoxy composites under Ar, (**a**) TG, (**b**) DTG.

The TGA results for pure epoxy and HF-Ti_3_C_2_T_x_/epoxy composites under Ar are shown in Figure 5, and the residual masses at 800 °C are listed in Table 1. Within the temperature range of 50–800 °C, the weight loss behavior of all the samples demonstrates a similar trend. The weight loss of epoxy starts at ~350 °C, which becomes insignificant when temperature is higher than ~550 °C, and the residual mass of epoxy at 800 °C is 8.3%, as listed in Table 1. For epoxy composites, the weight loss starts at lower temperature than that of pure epoxy, and the onset degradation temperature generally decreases as the content of HF-Ti_3_C_2_T_x_ filler increases. The fall in thermal stability was confirmed by both DTG curves in Figure 5b and the temperature for 5% of material degrades, as listed in Table 1. Similar thermal behavior of epoxy composites was also observed by Zhang et al. [31]. Such phenomenon can be attributed to the finely dispersed thermally conductive Ti_3_C_2_T_x_ fillers, which lead to more rapid thermal response of the composites to the temperature rise. However, it is also possible that the introduction of HF-Ti_3_C_2_T_x_ may accelerate the degradation of epoxy matrix by some chemical mechanism, which would need further investigations. Table 1 also listed the calculated residual mass of HF-Ti_3_C_2_T_x_/epoxy composites, and the relative deviation between the experimental and calculated values (Supplementary Equation (S2)) is within 1–8%, indicating the compositions of the as-prepared composites well match the original feed ratios of HF-Ti_3_C_2_T_x_ and epoxy components. 

**Figure 6:**
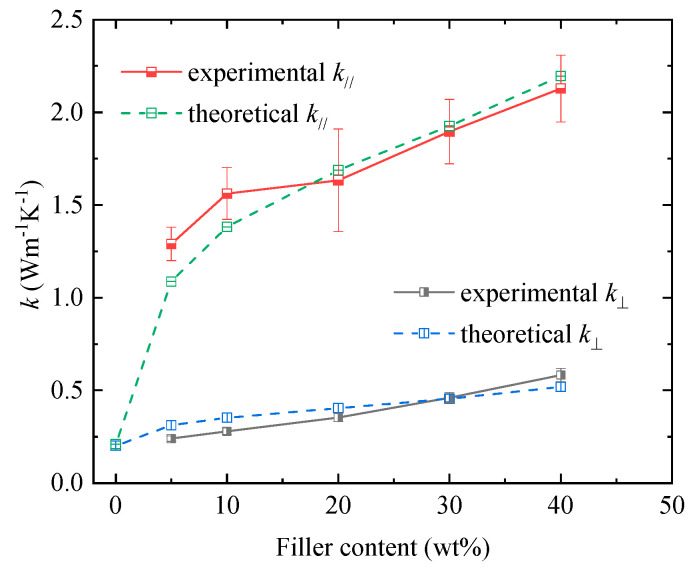
In-plane and through-plane thermal conductivities of HF-Ti_3_C_2_T_x_/epoxy composites, in which the theoretical ones are simultaneously calculated by Chen model [36,37].

Figure 6 shows the measured thermal conductivity results of the prepared composites, which are highly anisotropic since the in-plane thermal conductivity (*k*_//_) is much higher than the through-plane one (*k*_⊥_). Both *k*_⊥_ and *k*_//_ increase with the filler content, with *k*_⊥_ reaching 0.583 Wm^−1^K^−1^ when the filler content increases to 40 wt% (21.3 vol%), an enhancement of 2.92 times as compared with epoxy of 0.2 Wm^−1^K^−1^. In comparison, *k*_//_ achieves 1.29 Wm^−1^K^−1^ when filler content is only 5 wt% (2.1 vol%), and further increases to 2.13 Wm^−1^K^−1^ as filler content increases to 40 wt%, which are 6.45 and 10.65 times of that of epoxy, respectively. 

The enhanced but anisotropic thermal conductivity of epoxy composites can be reasonably related to the 2D structure of HF-Ti_3_C_2_T_x_ fillers and their distribution in the epoxy matrix. It is already known that 2D fillers, such as graphene [24,32] and boron nitride(BN) [33,34], can more effectively enhance composite thermal conductivity along the lateral direction of the platelet-shaped fillers, and this enhancement is more pronounced when the fillers tend to align in the composites, resulting in highly anisotropic thermal conductivity of the composite. In this work, the HF-Ti_3_C_2_T_x_/epoxy composites were prepared by cast molding. The composite fluid containing 2D fillers was continuously stirred at high speed for more than 2 h and then slowly poured into shallow mold where it gradually cured, and the resulting filler distribution is generally aligned. Su et al. [35] applied very similar procedures to prepare 2D graphite/epoxy composites, and they also obtained aligned filler distribution in the in-plain direction. Therefore, the aligned filler distribution might be attributed to the shear and centrifugal force in stirring. In addition, the gravity and viscosity force might also influence filler distribution. As a result of the aligned filler distribution, the in-plane thermal conductivity is effectively improved even with very small amount of filler, such as the 6.45 times enhancement of in-plane thermal conductivity achieved by 5wt% HF-Ti_3_C_2_T_x_ fillers. When the filler content increases, the distance between fillers reduces and the connection between fillers increases, as observed by SEM images in Figure 2. Consequently, heat conducting chain/network gradually forms as filler content increases, which contributes to the improved composite thermal conductivity. However, percolation phenomenon (i.e., sharp increase in thermal conductivity) is not observed in Figure 6, even when the filler content increased to 40 wt%. This may be related to the relative limited aspect ratio (around 3~6) of the prepared micro-sized HF-Ti_3_C_2_T_x_ platelets, as compared with its derivants of ML-Ti_3_C_2_T_x_ nanoflake and FL-Ti_3_C_2_T_x_ nanosheets. In other words, if nano-sized ML-Ti_3_C_2_T_x_ or FL-Ti_3_C_2_T_x_ were used, a higher thermal conductivity might be expected, which will be studied in future work. 

Figure 6 also shows the calculated thermal conductivity results obtained by a specialized model which was formerly developed for 2D BN/polymer composites [36,37] and was recently applied to analyze the Ti_3_C_2_T_x_/epoxy composites. Briefly, this model takes into account the key parameters including filler orientation angle and filler aspect ratio (i.e., determined by lateral size and thickness), and simultaneously calculates *k*_⊥_ and *k*_//_ for a given filler content. In this work, the filler orientation angle and aspect ratio are estimated from SEM images (Supplementary Materials Section S3) to be ca. 20° and 3.5, respectively. By using these parameters, together with an estimated thermal conductivity of 100 Wm^−1^K^−1^ for Ti_3_C_2_T_x_ filler [28], the modeling results fit quite well with the experimental results in both through-plane and in-plane directions, as shown in Figure 6, confirming the highly anisotropic thermal conductivity results from the platelet shape of the 2D Ti_3_C_2_T_x_ filler and their close-to-horizontal distribution. 

**Figure 7:**
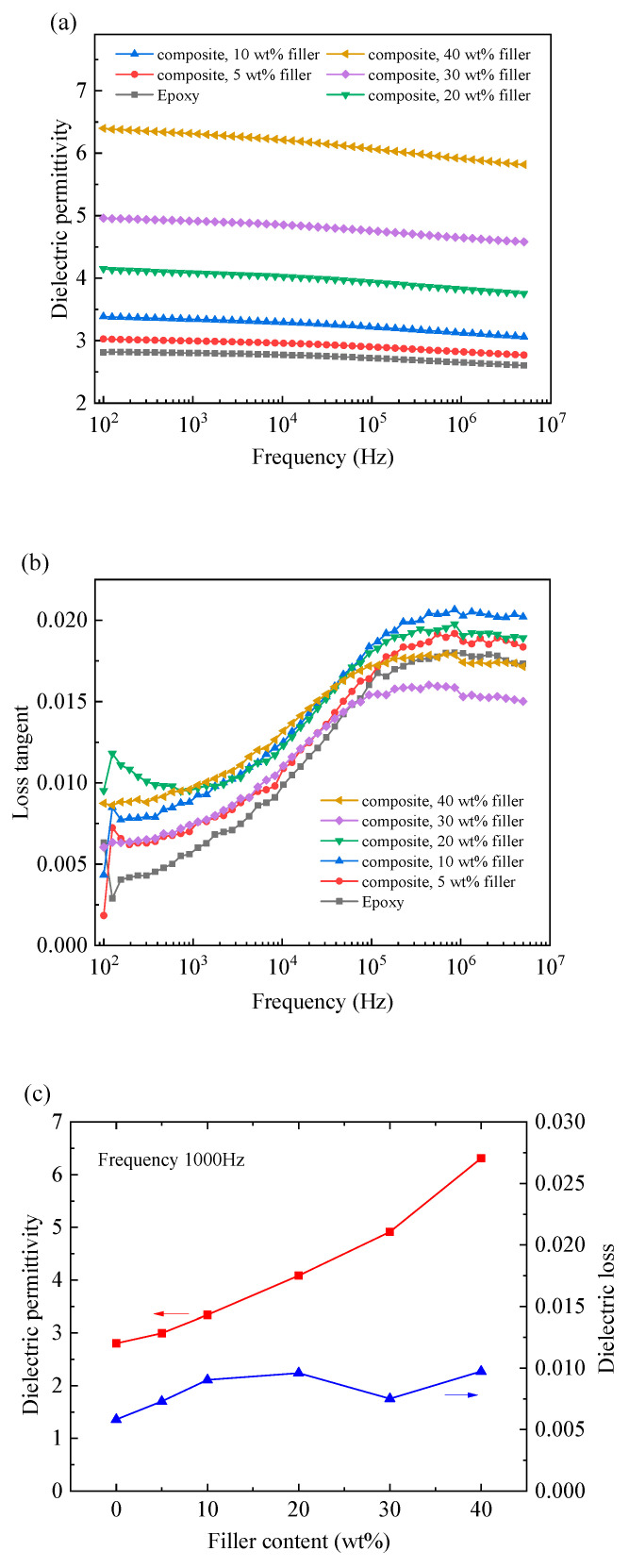
Frequency dependence of (**a**) dielectric permittivity and (**b**) dielectric loss of HF-Ti_3_C_2_T_x_/epoxy composites, and (**c**) variation of dielectric properties with the filler content at 10^3^ Hz.

Figure 7 shows the dielectric properties of the prepared HF-Ti_3_C_2_T_x_/epoxy composites which were measured at room temperature within the frequency range of 10^2^ to 5×10^6^ Hz. As shown in Figure 7a, the dielectric permittivity *ε* of each sample decreases slightly as the frequency increases. Similar phenomenon were also observed for epoxy composites [11,38,39], which can be attributed to the relaxation process [11,38], i.e., the decrease in dipolar polarization and interfacial polarization [39]. Moreover, the fact that the decrease of the dielectric permittivity with the frequency is quite smooth (i.e., dielectric permittivity decreases slightly with the increasing frequency) may reflect a uniform filler distribution in the epoxy matrix [11]. Figure 7a also shows that the dielectric permittivity of epoxy composite increases as the filler content increases. Take the data at 10^3^ Hz for example, as shown in Figure 7c, the dielectric permittivity increases to 6.3 when the filler content is 40 wt%, which is 2.25 times of pure epoxy (*ε* = 2.8). For other frequency, there is similar enhancement ratio since the variation curves are nearly parallel to each other. It is known that the dielectric permittivity of polymer composites can be improved by adding conductive fillers [4,39], which can lead to drastic increase in the vicinity of percolation threshold [4]. Therefore, the enhanced dielectric permittivity of epoxy composite in this work is induced by the conductive HF-Ti_3_C_2_T_x_ fillers. However, the achieved enhancement is limited since percolation is not attained even with 40 wt% of the micro-sized HF-Ti_3_C_2_T_x_ platelet fillers, which is consistent with the above analysis for thermal conductivity enhancement. From Figure 7b, the dielectric loss (i.e., loss tangent) generally increases with the increasing frequency and filler content, which is mainly due to relaxation process in polymer. The highest dielectric loss of about 0.02 appears at high frequency on the order of 10^6^. At 10^3^ Hz, the dielectric losses of all composites are within 0.01, as shown in Figure 7c.

## 4. Conclusions

In this work, epoxy composites were fabricated by using HF-Ti_3_C_2_T_x_ micro-platelet fillers, which can be efficiently and massively produced by direct HF etching of Ti_3_AlC_2_ powders. Both thermal and dielectric performance were improved by the addition of HF-Ti_3_C_2_T_x_ fillers. On the one hand, HF-Ti_3_C_2_T_x_ fillers effectively enhanced the thermal conductivity of epoxy composites. The through-plane thermal conductivity *k*_⊥_ achieved 0.583 Wm^−1^K^−1^ when the filler content was 40 wt% (21.3 vol%), an enhancement of 2.92 times as compared with pure epoxy. As for in-plane thermal conductivity k_//_, it reached 1.29 Wm^−1^K^−1^ when the filler content was only 5 wt% (2.1 vol%), and it further increased to 2.13 Wm^−1^K^−1^ when the filler content was 40 wt%, achieving the enhancements of 6.45 and 10.65 times, respectively. The enhanced but anisotropic thermal conductivity was attributed to the 2D structure of HF-Ti_3_C_2_T_x_ fillers and their distribution in the epoxy matrix, as observed by SEM observations. On the other hand, the conductive HF-Ti_3_C_2_T_x_ fillers also contributed to the dielectric permittivity of epoxy composite, and an enhancement factor of 2.25 was achieved by 40 wt% fillers. Meanwhile, the dielectric loss was also slightly increased, but all the measured results were within a limited value of 0.02. Therefore, our work has provided a promising way to make use of these HF-Ti_3_C_2_T_x_ micro-platelets as effective fillers to improve both the thermal and dielectric performances of epoxy composites.

## Figures and Tables

**Table 1:** Thermogravimetric data of HF-Ti_3_C_2_T_x_, epoxy and their composites.

Samples	Measured Residual Mass, (wt%)	Calculated Residual Mass (wt%)	Relative Deviation (%)	Temperature for 5 wt% of Material Degradation (°C)
HF-Ti_3_C_2_T_x_	92.3	-	-	-
Epoxy	8.3	-	-	364.3
HF-Ti_3_C_2_T_x_/epoxy composite, 5wt%	13.5	12.5	8	333.6
HF-Ti_3_C_2_T_x_/epoxy composite, 10wt%	17.8	16.7	7	347.5
HF-Ti_3_C_2_T_x_/epoxy composite, 20wt%	24.9	25.1	-1	335.5
HF-Ti_3_C_2_T_x_/epoxy composite, 30wt%	36.3	33.5	8	322.7
HF-Ti_3_C_2_T_x_/epoxy composite, 40wt%	45.2	41.9	8	326.6

## Supplementary Materials

The following are available online at https://www.mdpi.com/2073-4360/12/7/1608/s1.
