# Supplementary material for: Simultaneously Improved Thermal and Dielectric Performance of Epoxy Composites Containing Ti3C2Tx Platelet Fillers
Source: Polymers (Basel). 2020 Jul 19;12(7):1608. doi: 10.3390/polym12071608 (PMC7408071; doi:10.3390/polym12071608)
Supplement: Supplementary file 1 [file polymers-12-01608-s001.pdf]

Supplementary Materials

for

Simultaneously Improved Thermal and Dielectric Performance of Epoxy Composites Containing $Ti_3C_2T_x$ Platelet Fillers

Lin Chen[1,*], Yu Cao[1], Xuebo Guo[1], Ping Song[1], Kai Chen[2], Diansen Li[3], Jun Lin[4,**]

[1] MOE Key Laboratory of Power Station Energy Transfer Conversion and System, School of Energy Power and Mechanical Engineering, North China Electric Power University, Beijing 102206, China

[2] MOE Key Laboratory of Enhanced Heat Transfer and Energy Conservation, School of Chemistry and Chemical Engineering, South China University of Technology, Guangzhou 510640, Guangdong, China

[3] MOE Key Laboratory of Bio-Inspired Smart Interfacial Science and Technology, School of Chemistry, Beijing University of Aeronautics and Astronautics, Beijing 100191, China

[4] School of Renewable Energy, North China Electric Power University, Beijing 102206, China

* Correspondence: chenlin@ncepu.edu.cn, +86-10-6177-3372; jun.lin@ncepu.edu.cn, +86-10-6177-2185

S1. Three forms of $Ti_3C_2T_x$ materials

According to our previous study on $Ti_3C_2T_x$ MXene films[1], there are roughly three forms of $Ti_3C_2T_x$, including $Ti_3C_2T_x$ micro-platelets/particles resulted from hydrofluoric acid (HF) etching of Ti_3AlC_2 (herein, termed as HF-$Ti_3C_2T_x$) and its derivants of un-delaminated multi-layer $Ti_3C_2T_x$ nanoflakes (ML-$Ti_3C_2T_x$) and delaminated few-layer $Ti_3C_2T_x$ nanosheets (FL-$Ti_3C_2T_x$).

Figure S1 shows the relation and difference among the three forms of $Ti_3C_2T_x$. The filler used in this work is the non-exfoliated HF-$Ti_3C_2T_x$ micro-platelets/particles, which are produced by direct HF etching of Ti_3AlC_2 but without further DMSO intercalation and exfoliation by ultrasonication. Therefore, the thickness of the HF-$Ti_3C_2T_x$ is still on the order of micrometers, resulting in a relatively low aspect ratio. In Figure S1, the FL-$Ti_3C_2T_x$ is the MXene of which the thickness is on the order of nanometers and therefore has very high aspect ratio.

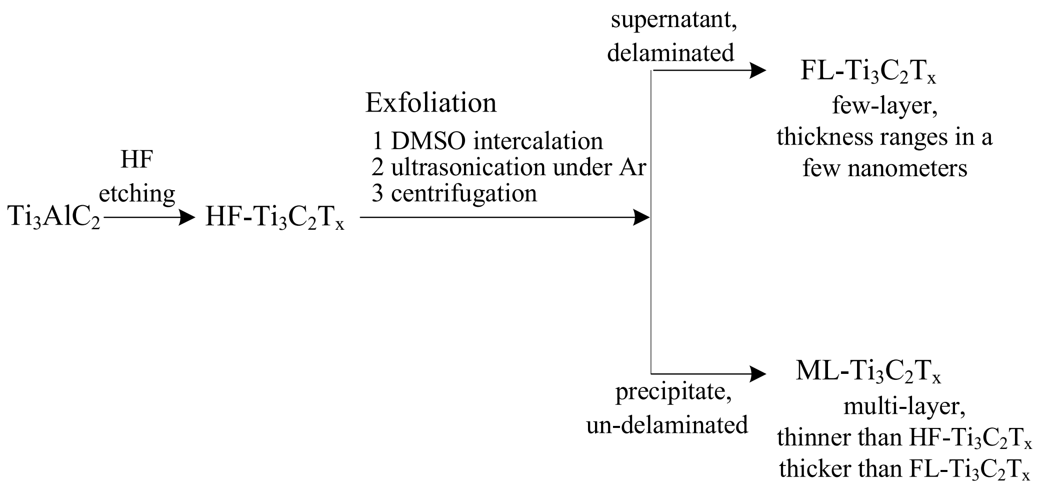

Figure S1. The process of manufacturing three forms of $Ti_3C_2T_x$

S2. Equations for calculating density and residual mass

The theoretical density is calculated by

$$\rho_c = \frac{1}{W_f/\rho_f + (1-W_f)/\rho_m} \quad \text{(S1)}$$

where W_f is the weight percentage of HF-$Ti_3C_2T_x$ filler, ρ_f, ρ_m and ρ_c are the densities of HF-$Ti_3C_2T_x$ filler, epoxy matrix and composite, respectively.

The theoretical residual mass, i.e., “Calculated residual mass” in Table 1, is calculated by

$$m_{cal} = W_f \cdot m_{mea,f} + (1-W_f)\cdot m_{mea,m} = W_f \times 92.3 + (1-W_f)\times 8.3 \quad \text{(S2)}$$

where W_f is the weight percentage of HF-$Ti_3C_2T_x$ filler, m_{cal}, $m_{mea,f}$ and $m_{mea,m}$ are the calculated residual mass, measured residual mass of HF-$Ti_3C_2T_x$ filler and measured residual mass of epoxy, respectively.

S3. Evaluation of orientation angle of filler

The images in Figure 2 mainly show the filler distribution in the matrix and the interface of filler and matrix. However, Figure 2 also shows the platelet fillers are more likely distributed along the in-plane direction, as indicated by the dashed lines for filler and filler chains in Figure 2b ~ 2f.

Figure S2 shows an example of estimating the orientation angle of filler. The composite specimen is adjusted to the horizontal position at low-resolution when taking SEM images, as shown in Figures S2a and S2b. In this way, the filler position observed at high-resolution image reveals its relation with the horizontal direction, namely, the in-plane direction, as shown in Figure S2c. A couple of orientation angles are measured, which are mainly in the range of 10 ~ 30°, and the average value of ca. 20° is used in the modeling.

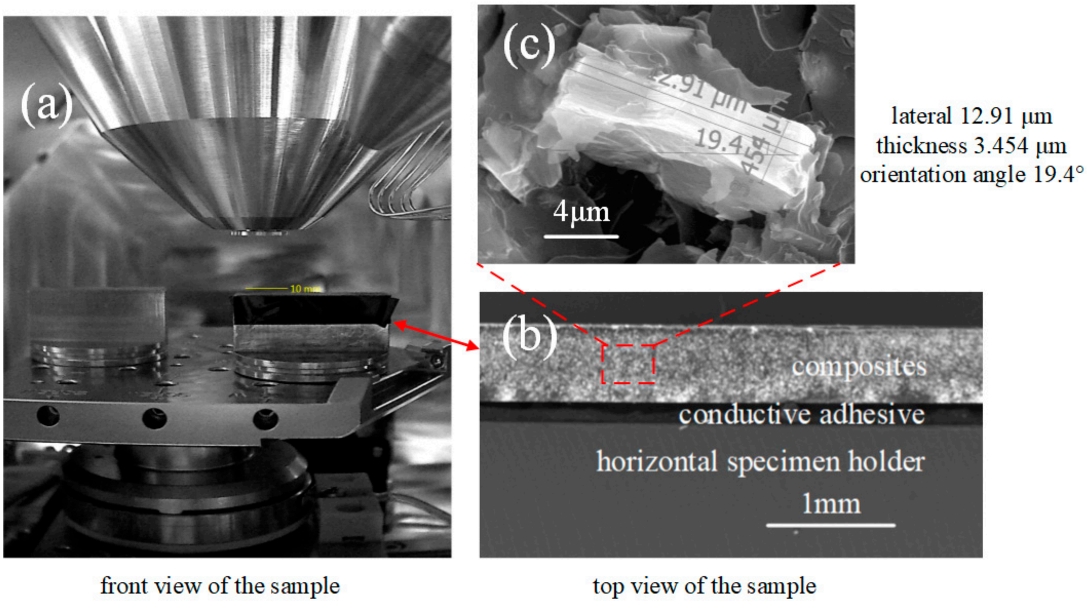

Figure S2. Demonstration of estimating orientation angle using SEM

S4. Mechanical properties of composites

Nanoindentation tests were performed by a Nano Indenter G200 (Keysight, USA) for epoxy and HF-$Ti_3C_2T_x$/epoxy composites containing 10wt%, 20 wt% and 30wt% fillers. Since the main topic of this paper is thermal and dielectric performance of HF-$Ti_3C_2T_x$/epoxy composites, the mechanical results is not included in the main content of manuscript. However, this information may be of interest when considering possible applications. The load-displacement results are shown in Figure S3. Based on the load-displacement results, the Young's modulus and hardness can be obtained, which are shown in Figure S4 and Figure S5, respectively.

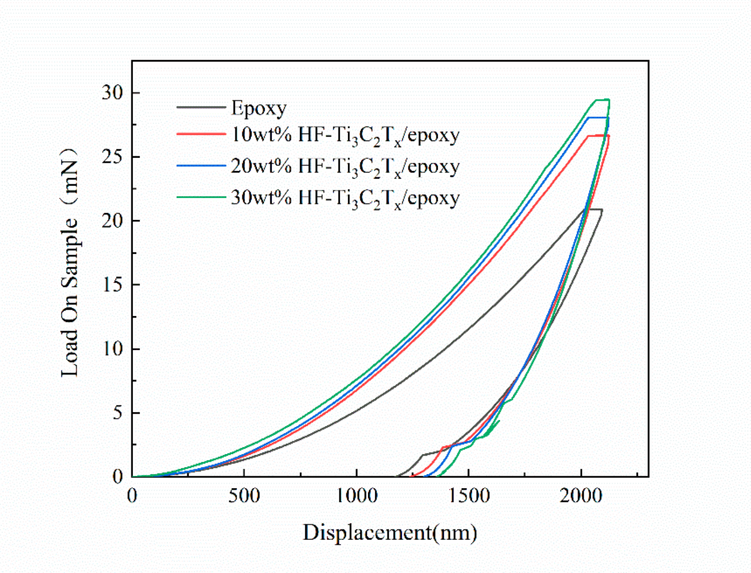

Figure S3 Load-displacement results in nanoindentation tests

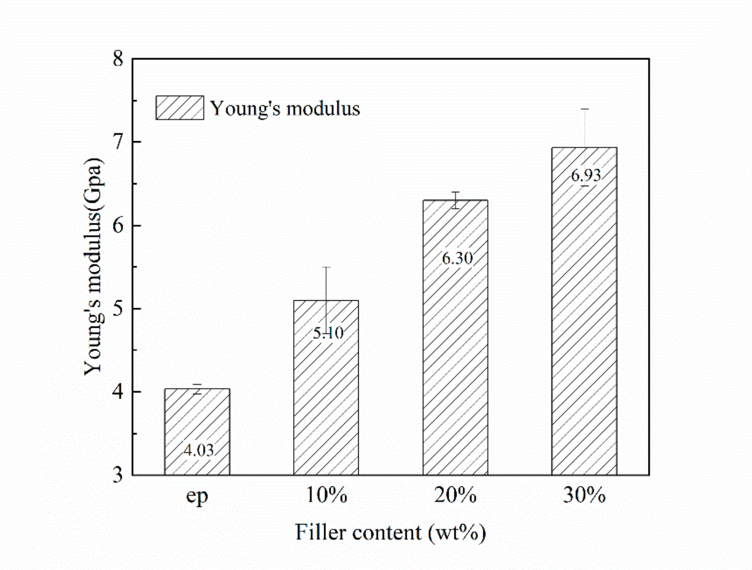

Figure S4 Young's modulus for epoxy and HF-$Ti_3C_2T_x$/epoxy composites

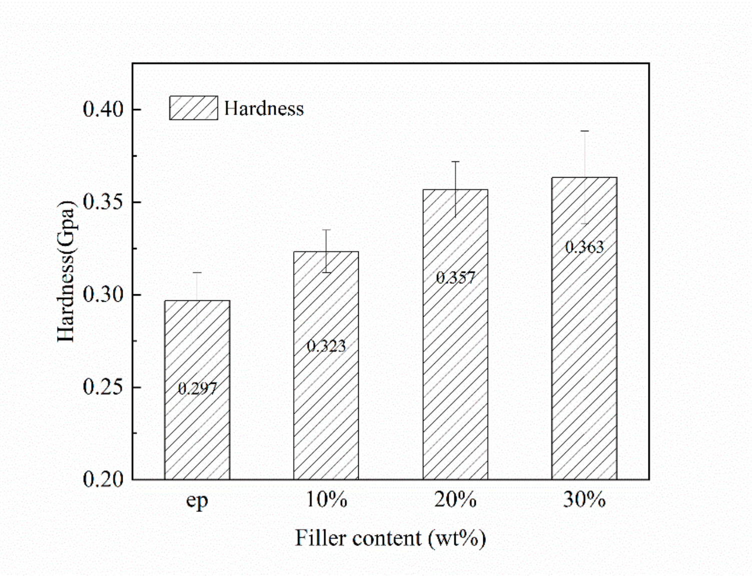

Figure S5 Hardness for epoxy and HF-$Ti_3C_2T_x$/epoxy composites

References

1. Chen, L.; Shi, X. G.; Yu, N. J.; Zhang, X.; Du, X. Z.; Lin, J., Measurement and Analysis of Thermal Conductivity of Ti3C2Tx MXene Films. *Materials* **2018,** 11, (9).
2. Aakyiir, M.; Araby, S.; Michelmore, A.; Meng, Q.; Amer, Y.; Yao, Y.; Li, M.; Wu, X.; Zhang, L.; Ma, J., Elastomer nanocomposites containing MXene for mechanical robustness and electrical and thermal conductivity. *Nanotechnology* **2020,** 31, (31), 315715.
